# Value appropriation in hepatitis C

**DOI:** 10.1007/s10198-021-01409-7

**Published:** 2021-12-02

**Authors:** Peter Lindgren, Sofia Löfvendahl, Gunnar Brådvik, Ola Weiland, Bengt Jönsson

**Affiliations:** 1 Department of Learning, Informatics, Management and Ethics, Karolinska Institutet, 171 77 Stockholm, Sweden; 2 The Swedish Institute for Health Economics, Lund, Sweden; 3 Department of Medicine, Division of Infectious Diseases, Karolinska Institutet and Karolinska University Hospital Huddinge, Stockholm, Sweden; 4 Stockholm School of Economics, Stockholm, Sweden

**Keywords:** Hepatitis C, Consumer surplus, Producer surplus, Pharmaceutical reimbursement, Cost-effectiveness, I18

## Abstract

**Background:**

In 2015, the Swedish government in an unprecedented move decided to allocate 150 million € to provide funding for new drugs for hepatitis C. This was triggered by the introduction of the first second generation of direct-acting antivirals (DAAs) promising higher cure rates and reduced side effects. The drugs were cost-effective but had a prohibitive budget impact. Subsequently, additional products have entered the market leading to reduction in prices and expansions of the eligible patient base.

**Methods:**

We estimated the social surplus generated by the new DAAs in Stockholm, Sweden, for the years 2014–2019. The actual use and cost of the drugs was based on registry data. Effects on future health care costs, indirect costs and QALY gains were estimated using a Markov model based primarily on Swedish data and using previous generations of interferon-based therapies as the counterfactual.

**Results:**

A considerable social surplus was generated, 15% of which was appropriated by the producers whose share fell rapidly over time as prices fell. Most of the consumer surplus was generated by QALY gains, although 10% was from reduced indirect costs. QALY gains increased less rapidly than the number of treated patients as the eligibility criteria was loosened.

**Conclusions:**

The transfer of funds from the government to the regions helped generate substantial surplus for both consumers and producers with indirect costs playing an important role. The funding model may serve as a model for the financing of innovative treatments in the future.

## Introduction

In the absence of treatment, patients with chronic hepatitis C infection develop a gradually worsening fibrosis of the liver which, with time, may lead to cirrhosis and an increased risk of hepatocellular cancer. Prior to the introduction of the second-generation direct acting antiviral agents (DAA) for the treatment of hepatitis C, physicians had to rely on ribavirin in combination with pegylated interferon (peg-IFN) with the possibility to add a protease inhibitor (boceprevir or telaprevir) for patients infected with the genotype 1 strain of the virus (triple therapy). Sustained virologic response (SVR), the main measure of efficacy, could be expected to reach 80% in patients also infected with genotype 1, but came at the cost of many severe adverse events [1]. The new generation of medicines have an efficacy of 90% with very few adverse events, but with the disadvantage of a higher price tag [2].

Not surprisingly, the cost-effectiveness of these new agents has received considerable attention with a large number of cost-utility analyses published the vast majority of which were conducted from a health care payer perspective [3]. In general, studies have indicated acceptable cost-effectiveness ratios in most instances with better cost-effectiveness ratios in more severely ill patients. Incremental cost-effectiveness ratios (ICERs) have been ranging from savings to below 50,000 USD per quality-adjusted life year (QALY) in most cases, with particularly encouraging results when compared with the older triple therapy including peg-IFN. Due to the substantial pool of patients eligible for treatment and the price tag of the new drugs, there were issues with short-term budget restrictions, leading to delays in use of the new direct antiviral (DAA) drugs even in cases when the new medicines were found to be cost-effective.

In Sweden, health care delivery is decentralized to 21 health care regions with large autonomy, including the right to levy taxes to finance their activities. Decisions on reimbursement of medicines administered at outpatient pharmacies are, however, taken at the national level (by TLV, the Dental and Pharmaceutical Benefits board). Although, there is a transfer of some funds from the national government to the regions, there is no direct link between a decision by TLV and changes to drug budgets at the regional level. Certain infectious diseases, including hepatitis C infection are considered a danger to the public and are covered by specific legislation requiring contact tracing and reporting of cases to the Public Health Agency for monitoring at the national level. Strategies for detection and treatment are managed at the regional level. There is currently no eradication strategy in place for hepatitis C.

In their first assessment, taking both cost-effectiveness as well as the potential budget impact into account, TLV limited reimbursement of the new drugs to mainly patients already suffering from severe consequences of the disease, specifically focusing on fibrosis stage 3 or 4 (4 meaning cirrhosis), but this still constituted a major financial hurdle for the regions. As a consequence, and possibly taking the legal classification of the disease into account, the national government made a unique decision in 2015 to transfer funds (approximately 150 million €) earmarked for these new drugs, some of which was intended to cover costs accrued in 2014 when the drugs where launched [4]. This agreement has then been extended annually with roughly 80–100 million €. Confidential rebates (implemented as refunds) were also agreed between TLV, the regions and the manufacturers through a process known as three-party negotiations. These negotiations were repeated as new products entered the market, allowing TLV to gradually loosen restrictions on the use of the drugs, first by including patients with fibrosis stage 2 in mid-2015 and then by removing all restrictions in 2018.

### Purpose and outline of the study

Ex ante cost-effectiveness studies are useful to provide information for pricing, reimbursement and use of new pharmaceuticals. Such studies compare costs and effectiveness in a defined indication compared to existing standard of care. The studies do not provide information about the total costs and benefits of the actual use of the drug for a defined period of time.

We studied the actual use and consequences of the introduction of DAAs in Sweden for the years 2014–19 in terms of health care costs, indirect costs, and health benefits. The total social surplus was estimated in a way that it is possible to get information about the distribution of costs and benefits created between different parties involved; patients, the health care provider (the regions), the government, and the companies selling the drug. The approach makes it possible to investigate how the surplus and its distribution have changed over time as prices and used for different indications has changed.

## Materials and methods

The social surplus *w* at a given output level *q* can be defined as

1 $$w\left(q\right)=z\left(q\right)+ \pi \left(q\right)$$

where *z* is the consumer surplus and $\pi$ the producer surplus (profits). To estimate the consumer surplus, it is necessary to determine: (a) the number of patients treated; (b) the downstream consequences on costs (which may be increased or decreased) and (c) the quantity and value of the health effects obtained. The producer surplus is the profits generated by substituting the costs of production and distribution. This surplus should pay for previous investments (sunk costs financed by previous profits or borrowing) in R&D to take the product to the market. The producer surplus can be greater or smaller than the investment in R&D, thus determining the success of the firm in developing new drugs. The producer surplus should also finance R&D investments that do not result in a new marketable product. To estimate the producer surplus, we therefore calculate the sales value minus the costs to the producer for production and distribution of the drug.

Data on the number of patients treated was obtained by extracting patients treated in the region of Stockholm from the Swedish registry for hepatitis C (InfCare Hepatitis) [5].

**Fig. 1:**
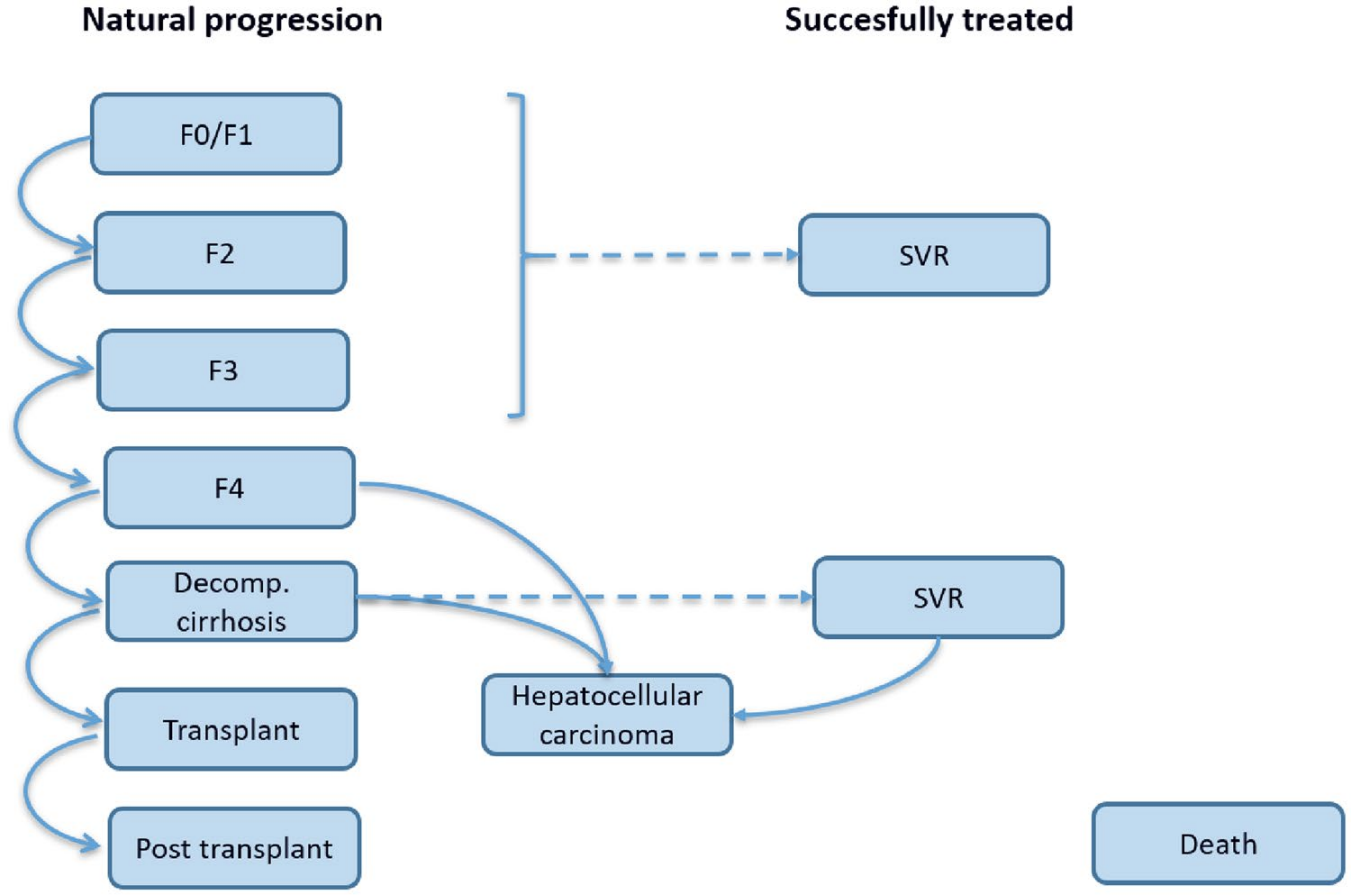
State transition diagram of the hepatitis C-model. Patients who do not progress remain in their current state. Transitions to death are possible from all states. F0–F4: fibrosis stage 0 to 4, stage 4 equals cirrhosis; SVR: sustained virologic response

To project future costs (discounted to present values at a rate of 3% in accordance with Swedish guidelines) we estimated a Markov model based primarily on Swedish data. A number of cost-effectiveness models in hepatitis C have been published in the past, all following a similar general framework [6, 7]. The main difference between the previously published models is the detail with which the early stages of disease is being represented. In our model (see Fig. 1) we opted for a division of fibrosis stages that capture the changes in recommendations made by TLV over time. Patients can progress from less to more severe fibrosis states, eventually suffering from liver failure with transplantation and a risk of developing hepatocellular cancer as potential consequences. Patients enter the model at the fibrosis stage they were at when they received treatment according to the hepatitis C registry and would then in annual cycles be followed for life. In later years (2018–2019), when restrictions on use only in more severe stages of the disease was lifted, there was some data missing on fibrosis stage. We assume that these would be milder patients and started them in the F0/F1 state of the model. As a contrafactual, we assumed that without the availability of second generation DAAs patients with genotype 1 would receive triple therapy while all other patients would receive dual therapy. When restrictions on reimbursement were eased, there was a large increase in the number of treated patients with mild disease (F0/F1). It is likely that these patients would not have been candidates for immediate treatment with the older agents. To take this into account, treatment with the older agents was only initiated when patients progressed to fibrosis stage F2 in cohorts initiating treatment 2018 or later.

The model was implemented in R version 4.0.1, utilizing version 0.9.4 of the heemod package [8, 9].

### Transition probabilities

A summary of the transition probabilities can be found in Table 1. The natural progression over fibrosis stage in untreated patients was derived from a meta-analysis by Thein al [10]. As, in the absence of antiviral therapy little can be done about the progression of the disease the progression rate can be assumed to be similar across geographies and this meta-analysis has indeed formed the basis of most previous models. Data on the risk of developing hepatocellular carcinoma (HCC) and need for transplantation once patients develop cirrhosis was derived through calibration from recently published data from a population based cohort of patients with cirrhosis in southern Sweden [11, 12].

**Table 1:** Transition probabilities used in the model

Input	Value	Comment/Source
Disease progression		
F1 to F2	0.085	Meta-regression of state transitions [10]
F2 to F3	0.120	Ibid
F3 to F4 (compensated cirrhosis)	0.116	Ibid
F4 to decompensated cirrhosis	0.039	Fattovich et al. [19]
Cirrhosis to HCC	0.047	Population-based Swedish cohort [12]
Decompensated cirrhosis to transplantation	0.038	Calibrated to match population-based Swedish cohort [15]
SVR (post cirrhosis) to HCC	0.014	Meta-analysis [18]
Mortality		
Decompensated cirrhosis	0.148	Calibrated to match population-based Swedish cohort [12]
Transplant—first year	0.190	European liver transplant registry [14]
Transplant—subsequent years	0.059	Population-based Nordic study [13]
HCC	0.530	Population-based Swedish cohort [12]
All other states	Age specific general mortality	Swedish life-tables [16]
Treatment effect		
SVR triple therapy (genotype 1 only)	80%	Based on literature review [1]
SVR dual therapy	70%	Ibid
SVR second generation DAA	96%	Observed in Swedish hepatitis C registry [17]

F0–F4: fibrosis stage 0 to 4, DAA: direct acting antiviral, HCC: hepatocellular carcinoma, SVR: sustained virologic response

Mortality rates for transplant patients were based on hepatitis C-specific data from the European liver transplant registry for the first year, and from a population-based cohort of Nordic patients for subsequent years [13, 14]. Nilsson and colleagues reported a median survival of 11 months in patients with cirrhosis diagnosed with HCC, which assuming an exponential risk corresponds to an annual probability of dying of 0.530 [12]. The same authors have also reported data on transplantation-free survival in patients with decompensated cirrhosis which we calibrated the model to match [15]. For all other health states, we assumed that mortality would be the same as in the age-adjusted general population [16].

Analysis of data from the national hepatitis C registry has shown that 96% of patients treated with current generation DAAs reach SVR [17]. No distinction was made between individual drugs in our analysis. They were treated as an aggregate both in terms of efficacy and costs. Patients treated with dual-therapy (genotypes 2–6) have been expected to achieve SVR in 50–80%, while the corresponding figure for patients treated with triple therapy (genotype 1 only) is in the 63–83% range [1]. We used a treatment effect of 70 and 80% for these two groups in the model. Successfully treated patients (irrespective of treatment regimen) have been reported to have a remaining risk of HCC which we based on a recent meta-analysis [18].

### Costs

Cost of treatment with second generation DAAs was based on public sales figures reported, adjusted with the reported refunds resulting from the three-party negotiation between TLV, the regions, and the manufacturers (see Table 2). For confidentiality reasons, these savings were only reported at the aggregate level for the DAAs as a group and we therefore used an average price per patients without distinguishing between the exact therapies given. It can be noted that the government contribution actually exceeded the cost to the regions in 2019, likely an effect of underestimating the predicted patient numbers to be treated.

**Table 2:** Cost of second-generation direct acting antivirals

Year	Total sales at list price (thousand 2019 €)	Government contribution to regions (thousand 2019 €)	Refund from rebates (thousand 2019 €)	Patients	Cost per patient after rebates (2019 €)
2014	63,418	68,278	–	1002	63,292
2015	152,431	87,334	22,042	2640	49,390
2016	141,214	89,059	40,668	2881	34,900
2017	84,654	86,779	24,780	2347	25,511
2018	204,055	103,570	165,077	6552	5949
2019	114,692	100,195	88,012	5162	5169

Source: The National Board of Health and Welfare, data on file; TLV [20]

Dual and triple therapy were assumed to be administered in accordance with the algorithms described by Liang et al. with prices based on official Swedish price lists [1, 21].

To estimate health care costs for inpatient care, ambulatory care and prescription drugs associated with disease progression, we matched patient level data from the hepatitis C registry to the National Patient Register (which contain data on inpatient admissions and outpatient visits) and the Swedish Prescribed Drug Register. We then conducted a regression analysis adjusted for age and sex to estimate the excess cost for more severe fibrosis stages compared to F0/F1 in the year prior to treatment initiation. We could see no increase in costs for states F2 and F3 (in fact it was somewhat lower, but we set this cost to 0 in the model) while patients in stage F4 hade numerically higher costs. We also extracted data on inpatient admissions for patients undergoing liver transplantation or being diagnosed with HCC and used the mean yearly costs during the 5 years following inclusion in the model. Lacking data on the costs associated with decompensated cirrhosis, we followed assumptions made in a previously published Swedish model for this health state [22].

To estimate indirect costs, we relied on the human capital method, obtaining days away from work for the patients extracted from the hepatitis C registry from the research data base at the Swedish Social Insurance Agency (Sw. Försäkringskassan). The relationship between work absenteeism and fibrosis stage was estimated through regression analysis as for direct costs, using F0/F1 as the reference state and then multiplied with average annual income including average employer and pension contributions taking labour force participation into account. Lacking data on decompensated cirrhosis, we conservatively assumed that this would be similar to F4. Indirect costs associated with HCC was based on estimated total indirect costs reported by Lundqvist et al. [23]. We had no data on patients undergoing transplantation. We assumed that these would have similar indirect costs as patients with HCC during the first year after the transplantation and that they then would fully return to the work force. As a sensitivity analysis, we also provide an estimate of indirect costs based on the friction cost method by adjusting indirect costs based on data from Pike and Grosse who have reported that among studies who have provided estimates of indirect costs related to absenteeism using both the human capital approach and friction cost approach, these differ by a factor of 5.6. [24]

It has previously been reported that patients treated with the older regimens incurred higher indirect costs in the year following treatment than patients treated with the second generation DAAs [25]. This cost was applied to the treated patients, adjusted for the proportion of patients being in working age.

All costs were inflated to 2019 € using the consumer price index from Statistics Sweden, and then converted to € using an exchange rate of 1 € = 10,26 SEK [26, 27]. A summary of the costs used as model inputs can be found in Table 3.

**Table 3:** Costs (2019 €) and utilities used in the model

Input	Direct costs		Indirect costs		Utility	
	Value	Comment/Source	Value	Comment/Source	Value	Comment/Source
Health state						
F0/F1	0	Regression on national registry data	0	Regression on national registry data	0.82	Pol et al. [28]
F2	0	Ibid	0	Ibid	0.78	Ibid
F3	0	Ibid	1302	Ibid	0.76	Ibid
F4	2028	Ibid	5500	Ibid	0.68	Average of reported studies [29]
Decompensated cirrhosis	7740	Based on assumptions made in [22]	5500	Assumed the same as for F4	0.54	Ibid
Transplant	93,514	National registry data	18,956	Assumed the same as for HCC	0.46	Pol et al. [28]
Post-transplant	8992	Ibid	0		0.80	Ibid
HCC	12,946	Ibid	18,956	Based on cost-of-illness [23]	0.51	Ibid
SVR	0		0		0.82	Assumed the same as F0/F1
Treatments						
Triple therapy	29,825	Liang et al. and official price lists [1, 21]	6600	[25]	− 0.021	One-time disutility [30]
Dual therapy	13,023	Ibid	8954	Ibid	− 0.014	Ibid
Second generation DAA		See Table 2			0	

F0–F4: fibrosis stage 0 to 4, DAA: direct acting antiviral, HCC: hepatocellular carcinoma, SVR: sustained virologic response

### Valuation of health outcomes

To estimate the value of the health benefits generated, we calculated QALY gained in the standard approach to cost-utility analysis. We used the typical threshold value utilized by TLV in their assessments (80,000 € per QALY, subsequently varied in sensitivity analyses) to estimate the value of these benefits [31]. The gained QALYs were estimated concurrently with costs using the model described above. Buchanan-Hughes et al. have recently published a review of utility weights in hepatitis C that was used to source inputs for the models [29]. Utility weights prior to cirrhosis was based on a cross-sectional survey conducted in France, Germany and the UK [28]. This study was also used for data on transplant patients and patients with HCC. For patients with cirrhosis, a number of studies have reported utility weights ranging between 0.77 and 0.41, with lower weights reported for decompensated cirrhosis. We used the mean values from the reported studies [29]. For patients obtaining SVR studies reported utilities in the 0.8–0.95 range, we assumed that they would have the same utility as patients in the F0/F1 state. Dual and triple therapy is known to be associated with a fairly heavy adverse event burden. We, therefore, followed assumptions made in a previous modelling study and applied a one-time disutility (which was 50% higher for patients on triple therapy) to patients undergoing treatment [30].

As a sensitivity analysis, we employed an alternative approach using directly elicited estimates of the willingness to pay for the treatment based on a previously published study which assessed this from different perspectives [32]. We used a willingness to pay 120,000 € for treatment which corresponds to an ex-post valuation from a social perspective.

### Producer surplus

The per patient sales value based on national statistics and adjusted for confidential discounts is presented in Table 2. Philipson and Jena have, based on price reductions after patent expiration, previously estimated the cost to the producer for HIV drugs to 15% of the sales value [33]. As there is considerable in-patent competition when additional drugs where launched in our case, we applied a slightly lower (14%) figure to the sales for the third year (roughly the midpoint of the interval) to get an estimate of the cost per patient, which was then applied throughout. This implies that the sales price minus rebates during 2019 is very close to the marginal cost. The assumption was subsequently tested in sensitivity analysis.

### Ethical considerations

The study was approved by the ethical review board in Stockholm (2017/2243-31). Matching of patient data was conducted by the National Board of Health and Welfare; the researchers only had access to anonymized data during the conduct of the study.

## Results

**Fig. 2:**
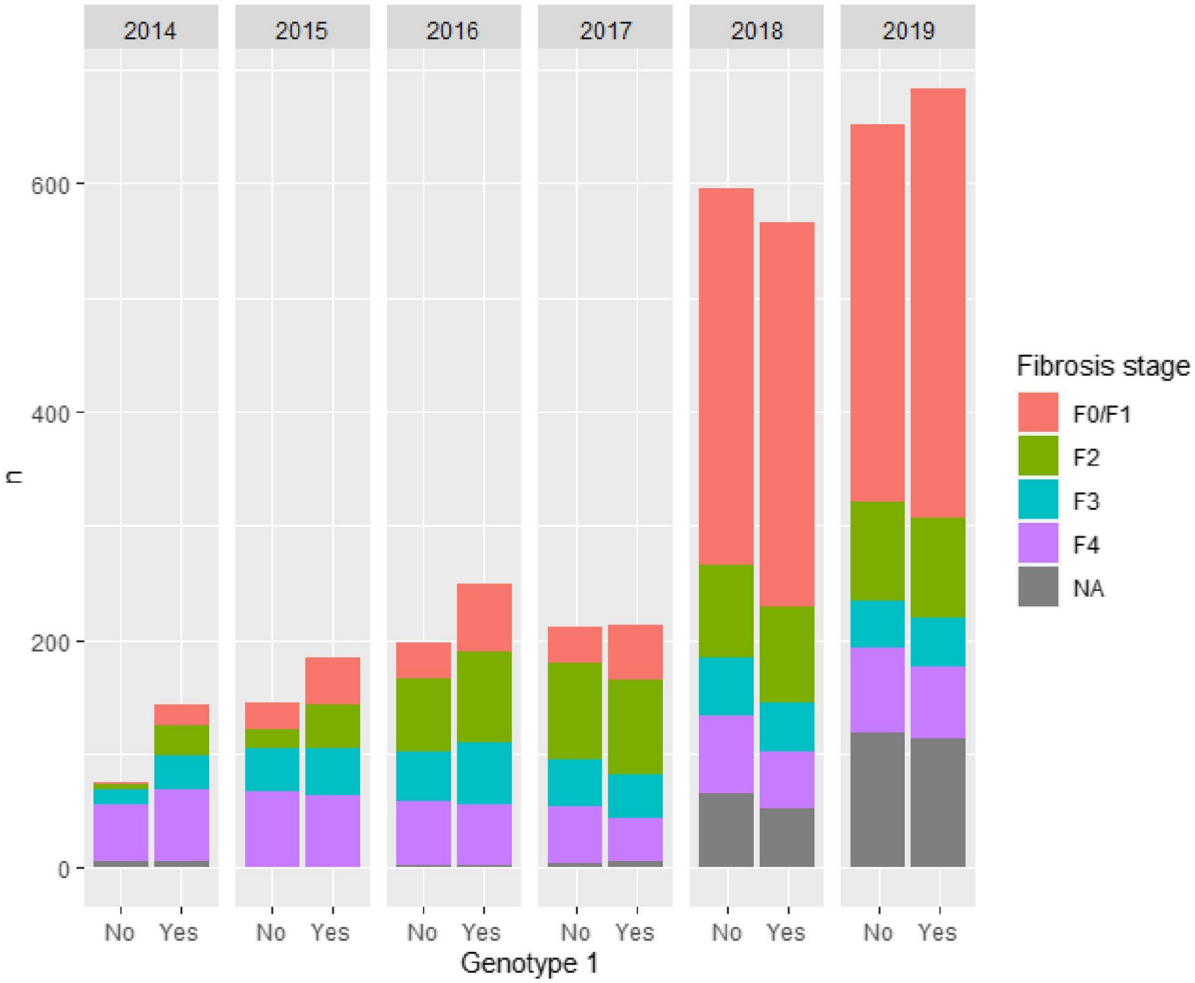
Number of treated patients by genotype and fibrosis stage

Figure 2 shows the number of treated patients by year in Stockholm, stratified by genotype and fibrosis stage. Except for the year 2014, the number of treated patients with genotype 1 (where patients are candidates to receive triple therapy) is roughly equal to the number of patients with all other genotypes combined. The consequence of TLV expanding reimbursement to all patients in 2018 can clearly be seen in the increased number of patients with milder disease treated in later years. It can also be noted that there is more missing information on fibrosis stage these years, which is a consequence of patients being treated outside the infectious diseases clinic when restrictions were lifted. Furthermore, the majority of these patients was judged to have mild disease from demographic data including age and aspartate aminotransferase to platelet ratio index (APRI) score. In 2019, 1334 patients or 0.57 per million inhabitants, were treated.

Table 4 shows the net lifetime cost for each of the treated cohorts, along with predicted health benefits in terms of life-years gained (LYG) and QALY as well as the ICERs. As more patients are treated over time, savings in health care costs and indirect cost increase. The effect on price reductions triggered by new market entrants is profound leading to overall cost savings (driven by reductions in indirect costs) from 2016. In 2018 and 2019 prices (including discounts) for the new DAAs were lower than the cost of the previously used double and triple therapy, leading to savings even on the treatment cost side. As a consequence, we can observe savings to both the health care system and society as a whole when considering the entire time period under study. These savings occurred more quickly among patients with genotype 1, explained by the higher cost of triple therapy compared to dual therapy.

**Table 4:** Net lifetime costs (thousand 2019 €) and health benefits in patients treated with second-generation DAAs by year of treatment initiation

	2014	2015	2016	2017	2018	2019	Total
Treatment cost	8137	8854	5498	1692	− 12,459	− 15,540	− 3819
Genotype 1	4618	3580	1253	− 893	− 9869	− 12,259	− 13,569
All other genotypes	3519	5273	4244	2585	− 2590	− 3,281	9750
Other healthcare costs	− 1251	− 1849	− 2223	− 2071	− 3890	− 4303	− 15,588
Genotype 1	− 634	− 758	− 932	− 749	− 1395	− 1644	− 6112
All other genotypes	− 617	− 1090	− 1291	− 1322	− 2495	− 2660	− 9475
*Direct costs*	6886	7005	3274	− 379	− 16350	− 19,843	− 19,406
Genotype 1	3984	2822	321	− 1642	− 11264	− 13,903	− 19,681
All other genotypes	2902	4183	2953	1263	− 5085	− 5940	275
Indirect costs	− 2625	− 3883	− 4738	− 4361	− 7562	− 8407	− 31,575
Genotype 1	− 1492	− 1847	− 2328	− 1891	− 3271	− 3850	− 14,680
All other genotypes	− 1133	− 2035	− 2410	− 2469	− 4290	− 4557	− 16,895
*Total costs*	4262	3122	− 1464	− 4740	− 23,911	− 28,250	− 50,981
Genotype 1	2492	975	− 2006	− 3534	− 14,536	− 17,753	− 34,361
All other genotypes	1769	2147	542	− 1206	− 9376	− 10,497	− 16,620
LYG	197	282	325	297	488	533	2123
Genotype 1	98	114	134	106	170	199	820
All other genotypes	99	169	190	192	318	335	1302
QALY	232	343	411	382	706	780	2855
Genotype 1	119	142	174	140	257	303	1134
All other genotypes	114	201	237	242	449	477	1721
Cost per QALY	18,337	9112	Dominance	Dominance	Dominance	Dominance	Dominance
Genotype 1	21,028	6888	Dominance	Dominance	Dominance	Dominance	Dominance
All other genotypes	15,536	10,677	2286	Dominance	Dominance	Dominance	Dominance

DAA: direct acting antiviral, LYG: life-years gained, QALY: quality-adjusted life-years

**Fig. 3:**
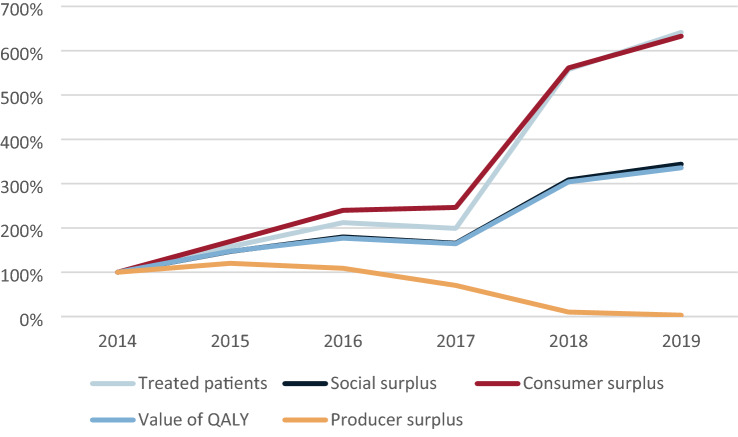
Relative change in the social surplus and its components compared to 2014

Table 5 shows the social surplus generated over time with Fig. 3 showing the relative growth of the social surplus and some of its components. It can be noted that saved indirect costs make up almost 10% of the generated social surplus and that the producers appropriated 15% of the generated social surplus during the studied period. The consumer surplus follows the development of the number of treated patients closely, despite milder patients being treated in later year and the value of the generated QALYs therefore, growing at roughly half the pace. The social surplus is growing at about half the pace of the generated consumer surplus, driven by the fact that the producer surplus is shrinking despite a larger number of patients being treated which is due to reduction in prices. This reduction in prices leads to offset in costs in later years as more expensive treatment options are being replaced.

**Table 5:** Social surplus and its components (thousand 2019 € per million inhabitants)

	2014	2015	2016	2017	2018	2019	Total
Treated patients^a^ (*n*)	208	328	441	414	1160	1334	3885
Social surplus	11,296	16,591	20,302	18,702	34,838	38,851	140,581
Consumer surplus	6114	10,364	14,656	15,059	34,312	38,690	119,194
Values of QALYs	7932	11,696	14,031	13,037	24,111	26,638	97,444
Direct costs	2938	2988	1397	− 162	− 6975	− 8465	− 8279
Indirect costs	− 1120	− 1656	− 2021	− 1860	− 3226	− 3587	− 13,471
Producer surplus	5183	6228	5647	3643	526	161	21 387
Share of social surplus (%)	45.9%	37.5%	27.8%	19.5%	1.5%	0.4%	15.2%

QALY: quality-adjusted life-years

^a^In the county of Stockholm, 2.3 million inhabitants

Table 6 shows the results from the sensitivity analyses. Variations in how QALYs are valued within the ranges tested here translates to fairly small changes in the overall results, while switching to an alternative valuation method has a larger impact on the size of the consumer surplus. Reducing the assumption about the share of the price making up the cost of production for the producer by up to two thirds did not influence the results greatly. Reducing the effect of the comparator treatments in line with the expected lower bounds reported by Liang did have a fairly large impact compared to our base case, which is somewhat close to the upper bounds; a shift in this direction did therefore not have a meaningful impact.

**Table 6:** Sensitivity analyses: total surplus generated 2014–2019 (2019 €, per million inhabitants)

	Social surplus	Consumer surplus	Producer surplus	Producer share of surplus (%)
Base case	140,581	119,194	21,387	15.2
Direct elicitation of WTP	242,027	220,641	21,387	8.8
Value of QALY				
30,000 €	72,588	51,201	21,387	29.5
60,000 €	116,220	94,833	21,387	18.4
100,000 €	164,942	143,555	21,387	13.0
Producer costs				
10%	142,894	119,194	23,701	16.6
5%	145,787	119,194	26,593	18.2
Friction costs	129,516	108,129	21,387	16.5
Comparator effect				
Lower bound of SVR^a^	240,492	219,105	21,387	8.9
Higher bound of SVR^b^	132,724	111,337	21,387	16.1

WTP: willingness-to-pay, QALY: quality adjusted life year, SVR: sustained viral response

^a^SVR 0.50 and 0.61 for dual and triple therapy respectively

^b^SVR 0.70 and 0.83 for dual and triple therapy respectively

## Discussion

Our analyses indicate that the introduction of the modern DAAs resulted in the generation of a substantial social surplus of which the consumers obtained the largest part, primarily due to health improvements resulting in improved survival and quality of life. During the period under study, between 8 and 18% of the value was appropriated by the producers depending on assumptions about the cost of production and the valuation of health benefits, with our central estimate being 15%. This can be compared with the on-brand period for simvastatin where the producer was estimated to appropriate 43% of the value based on similar methodology [34]. Interestingly, this share is the same as what was observed during the first year of DAA use in the present study, prior to the introduction of additional agents on the market. In contrast to the situation for statins, where the introduction of additional medicines such as atorvastatin and rosuvastatin had negligible effect on prices, Swedish payers engaged heavily in price negotiation as part of the three-party deliberation system now in place. As a consequence, in-patent competition caused a greater share of the social surplus to be appropriated by the payer, which is consistent with theoretical models of surplus distribution [35]. The notion that payers have a stronger bargaining position in this particular case is also consistent with findings from interviews conducted in other European countries [36]. In the long run, the results from the study on simvastatin indicates that post patent expiration the producer appropriates 1–2% of the surplus, which is in line with what we can observe in the last years of our observation period. This indicates that producers have been willing to trade price for volume at almost generic levels. Anecdotally, this seems like a unique situation. It is potentially due to the fact that we are dealing with a limited stock of patients, and that missing out at a given iteration in the negotiations would mean to risk no sales at all in the long run.

In addition to the health benefits generated by a very efficacious treatment strategy with cure rates above 90%, the consumer surplus is also driven upwards by the reduction in prices in later years where the prices of the new agents fall below those of the previous treatment options by a substantial margin. In addition to this, and a larger factor in terms of absolute contribution to the consumer surplus generated during the period, is savings in indirect costs. Indirect costs, making up almost 10% of the social surplus in the base case, have largely been omitted in studies of hepatitis C [37], including economic evaluations of DAAs [3]. Our analysis shows that these costs are an important part of the generated value.

Earlier studies of the cost-effectiveness of treatments in this field have shown that new interventions often have poor cost-effectiveness initially, but that this rapidly improves over time as the understanding of how to use the drug optimally increases which leads to both reduced costs and better health outcomes [22]. In our case, the great effect of price reductions takes the overhand when considering the value generated.

There are of course a number of methodological limitations to this work. As a counterfactual, we assumed that the patients not receiving treatment with the second generation DAAs would receive treatment with the previous generation of treatment. It is possible that a number of these patients would remain untreated or have their treatment postponed into the future due to the adverse event profile. Some patients may also have received treatment previously but remained unresponsive. It is likely that a smaller fraction of these patients would be successfully treated. In both these cases, we would likely underestimate the health benefits of treatment, and therefore underestimate the societal value generated.

Like most models evaluating the economics of the second generation DAAs, we did not take any transmission effects into account. This represents an underestimation of the value of the drugs which may be more important in the later years of our analysis where a wider group of patients are being treated. However, such a study must also explicitly take into account the risk of re-infection, and how that can be managed. Again, this would likely lead to an underestimation of the benefits of the new agents.

TLV estimates may not represent a true societal value of the health outcomes; in fact it is quite likely that threshold values utilized by TLV and similar agencies differ significantly from the social valuation depending on how these threshold values have been established. In the early days of decision making at TLV, there was some concordance between the threshold values seemingly applied by TLV and those more formally determined in the road sector. However, while the latter have been revised over time, the thresholds have remained stable or been adjusted based on other factors (such as rarity). It has been argued, based on recent data from the road sector, that the threshold value ought to be higher. In this case, the estimates of social surplus generated would be underestimated. Utilizing the directly elicited willingness-to-pay estimates may be a closer estimate of the societal valuation, but may omit some health consequences of hepatitis C that were not captured in the elicitation. It is also difficult to capture the time aspects of a slowly progressing disease like hepatitis C with this type of approach. Reliable data on the costs to the producer of providing the medicine is difficult to obtain. We relied here on previous estimates based on HIV drugs, but these may not be directly applicable. Indeed, applying this proportion to the price at launch in Sweden would results in implicitly assuming that medicines were provided at a loss in later years which does not seem feasible. Our sensitivity analysis indicated that even sharp reductions in this input did not have a major impact on the results, but additional research in this field would be useful for future studies.

By using a regression approach to estimate the costs associated with more severe fibrosis stages, we omit cost items that are similar across stages, for instance for monitoring of the disease. The effect of this is likely to be quite small: previous models have estimated one visit per year [22]. By relying on national registry data in this analysis, we also omit resource items that are not captured in the registries, notably primary care costs which may lead to a slight underestimation of the overall costs.

This analysis constitutes an after-the-fact analysis of the impact of the introductions of the second generation DAAs in a Swedish setting. As such, is relies on drug utilization data from Sweden (or more accurately Stockholm, which is taken as a proxy for the country as a whole). It may, in the general sense, be taken as indicative for countries where uptake of the new medicines has followed a similar pattern but the degree to which the specific are applicable is dependent on several factors. The three most important considerations would likely be: (1) the development of prices of the new DAAs over time. As we have illustrated here, there was a rapid erosion of prices over time. The situation is likely to have been similar in many countries as the fairly large number of medicines competing for a limited prevalent pool of patients gave payers increased bargaining power. Due to the extensive use of confidential rebates that are generally part of these negotiations, it is however hard to ascertain how well payers in different countries took advantage of this. (2) Indirect costs would in part be driven by labour market dynamics. Certain decision makers, notably in the Netherlands, Canada and Germany prefer the use of the friction cost method to value indirect costs as opposed to the human capital approach that we have employed here. We would agree with Johannesson and Karlsson that consistently applying the friction cost method would mean that the method should also be applied in the estimation of direct costs. [38] However, adjusting the savings in indirect costs estimated here by this factor, the consumer surplus would be reduced by 10% during the studied period and the producer share of the surplus increases from 15.2 to 16.5%, a very small effect. (3) Most importantly, the size of the consumer surplus is strongly linked on the valuation of the health benefit, in this case the value of a QALY. The societal valuation of a QALY may well vary between countries, although it is debatable if it in fact values as much as the threshold values employed by different HTA bodies do.

### Policy implications

The issue of affordability in contrast to cost-effectiveness have received considerable attention in the debate recently [39]. Indeed, hepatitis C has been raised as a case in point with for instance Pearson arguing that Sovaldi upon launch was cost-effective, but apparently not affordable [40]. This seems to apply for the reasoning in Sweden as well, with it being understood that the initial restrictions on use was at least in part due to affordability issues. Even with these restrictions, the national government found it advisable to step in with additional funds to support the regional payers and thereby facilitating access to these medicines. This can be seen as a successful initiative as significant surplus was generated even prior to price competition reduced the cost of treatment considerably. The consequences of this would extend beyond the health care system with savings for instance in the social insurance system which is managed nationally. We would argue that a similar model could be used in other areas where similar problems could arise, in particular curative treatments where a stock of patients are eligible for treatment or where there are significant savings downstream but short-term budget barriers that may prevent these being realizable, for instance in the advanced therapeutical medicinal products (ATMP) space. A major difference would be the need for additional measure to manage uncertainty around the long-term effects and therefore, the value realized in other fields. In the case of hepatitis C this was never an issue as there was almost complete agreement about the efficacy of the drugs based on data from the clinical trials. This could be addressed using outcomes-based on conditions to reduce the decision-making uncertainty.

## Conclusion

Through a targeted transfer of funds from the Swedish national government to the payers at regional level uptake of new drugs for hepatitis C generated substantial surplus on both the consumer and producer side despite questions about affordability, with costs outside of the health care system playing an important role. In-patent competition, subsequently further increased the consumer surplus by driving down prices below the prices of previous generations of medicines, leading to shrinking producer surplus over time. The funding model may serve as a model for the financing of innovative treatments in the future.

## Data Availability

No.
